# Supplementary material for: Endothelial Semaphorin 3F Maintains Endothelial Barrier Function and Inhibits Monocyte Migration
Source: Int J Mol Sci. 2020 Feb 21;21(4):1471. doi: 10.3390/ijms21041471 (PMC7073048; doi:10.3390/ijms21041471)
Supplement: Supplementary file 1 [file ijms-21-01471-s001.pdf]

International Journal of *Molecular Sciences*

MDPI

Supplemental Figures

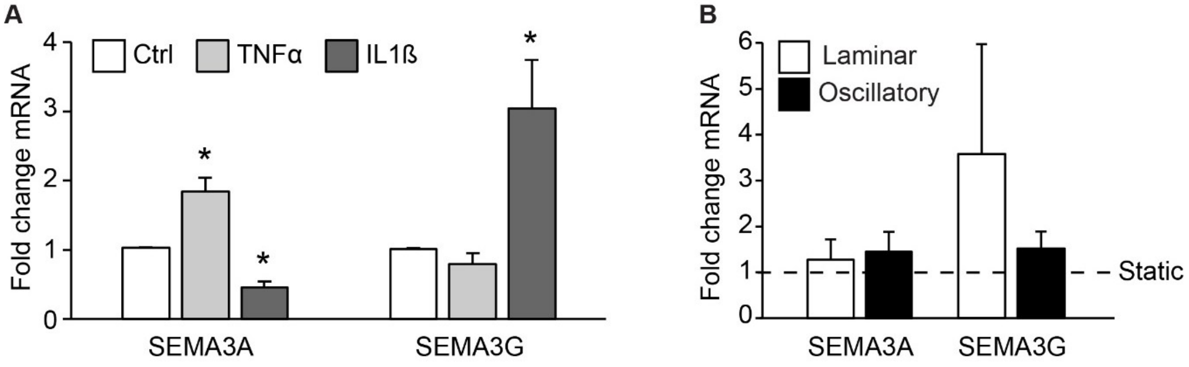

Supplemental Figure S1. Regulation of SEMA3A and SEMA3G expression by inflammatory factors or shear stress. (**a**) SEMA3A and SEMA3G expression in HUVECs stimulated with the inflammatory factors, TNFα (10 ng/ml) or IL1β (20 ng/ml) for 24 hours. Results are presented relative to untreated cells. Mean ± SEM of n=3; *p<0.05. (**b**) Quantitative qPCR analysis of SEMA3A or SEMA3G mRNA isolated from HUVECs cultured under laminar or oscillatory flow conditions (10 dyn/cm^2) for 7 days compared to static culture conditions. Results are presented relative to static cultured cells, set as 1. Mean ±S EM of n=3.
